# Amelioration of Cisplatin-Induced Ototoxicity in Rats by L-arginine: The Role of Nitric Oxide, Transforming Growth Factor Beta 1 and Nrf2/HO-1 Pathway

**DOI:** 10.31557/APJCP.2020.21.7.2155

**Published:** 2020-07

**Authors:** Remon S Estfanous, Walaa S Elseady, Ahmed M Kabel, Rasha A Abd Ellatif

**Affiliations:** 1 *Anatomy and Embryology Department, Faculty of Medicine, Tanta University, Tanta, Egypt. *; 2 *Pharmacology Department, Faculty of Medicine, Tanta University, Tanta, Egypt. *; 3 *Department of Clinical Pharmacy, College of Pharmacy, Taif University, Taif, Saudi Arabia. *

**Keywords:** Cisplatin, ototoxicity, L-arginine, inflammation, cancer

## Abstract

**Background:**

Cisplatin is an alkylating agent that inhibits DNA replication and interferes with proliferation of cancer cells. However, the major limiting factor for its use is the possible development of adverse effects, including ototoxicity. Up till now, the mechanisms of this ototoxicity remain poorly understood. However, induction of oxidative stress and activation of the inflammatory cascade were suggested as contributing factors.

**Purpose:**

The aim of this study was to explore the effect of L-arginine on cisplatin-induced ototoxicity in rats.

**Methods:**

Thirty male adult Wistar rats were divided into three equal groups as follows: control group; cisplatin group and cisplatin + L-arginine group. Auditory brainstem response (ABR), tissue oxidative stress parameters, total nitrate/nitrite, nuclear factor (erythroid-derived 2)-like 2 (Nrf2)/heme oxygenase-1 (HO-1) content, transforming growth factor beta 1 (TGF-β1), tumor necrosis factor alpha (TNF-α) and interleukin 15 (IL-15) were assessed. Also, the cochlear tissues were subjected to histopathological and electron microscopic examination.

**Results:**

Administration of L-arginine to cisplatin-treated rats induced significant decrease in the average ABR threshold shifts at all frequencies, tissue TGF-β1, TNF-α and IL-15 associated with significant increase in tissue antioxidant enzymes, total nitrate/nitrite and Nrf2/HO-1 content compared to cisplatin group. Also, pretreatment of cisplatin-injected rats with L-arginine induced significant improvement of the histopathological and electron microscopic picture compared to cisplatin group.

**Conclusion:**

L-arginine may serve as a promising therapeutic modality for amelioration of cisplatin-induced ototoxicity.

## Introduction

Cisplatin is one of the platinum-containing chemotherapeutic agents that are widely used for the treatment of various types of malignancies including testicular, ovarian, breast and bladder carcinoma (Dasari and Tchounwou, 2014). However, the major limiting factor for the use of cisplatin in cancer therapy is the possible development of serious adverse effects such as myelosuppression, nausea, vomiting, neuropathy, nephrotoxicity and ototoxicity (Achkar et al., 2018). Cisplatin administration usually leads to loss of the inner and outer hair cells together with irreversible damage of the spiral ganglion neurons resulting in irreversible hearing loss (Sheth et al., 2017). These harmful effects may be attributed to induction of oxidative stress with increased production of reactive oxygen species (ROS) together with affection of the inflammatory cascade which were proven to play an important role in the cochlear damage induced by cisplatin (Rybak et al., 2019).

Transforming growth factor beta 1 (TGF-β1) was reported to play a key role in the pathogenesis of cisplatin-induced ototoxicity (Gonçalves et al., 2013). TGF-β1 is expressed in both epithelial and mesenchymal tissues of the cochlea and is involved in otic capsule formation, spiral ganglion formation and survival of the hair cells (Murillo-Cuesta et al., 2015). Warnecke et al., (2019) reported that TGF-β1 is a master regulator of the immune response in several tissues, including the cochlea of the inner ear. Early after tissue damage, TGF-β1 was found to modulate the expression of adhesion molecules and induce chemotaxis and activation of leukocytes which in turn secrete large amounts of the proinflammatory cytokines including tumor necrosis factor alpha (TNF-α) and interleukin 15 (IL-15) (Hara et al., 2017). An early increase in the expression of TGF-β1 was reported during cochlear damage induced by aminoglycosides followed by down-regulation as the inflammatory response resolves. This may support the potential role of TGF-β1 in the pathogenesis of drug- induced cochlear toxicity (Murillo-Cuesta et al., 2015). Thus, targeting TGF-β1 may represent a potential hope for amelioration of cisplatin-induced ototoxicity (Schacht et al., 2012). 

Recent reports suggested that nitric oxide (NO) may play a significant role in protection against cisplatin-induced ototoxicity (Jamesdaniel et al., 2016). Wink et al., (1997) found that nitric oxide donors may inhibit the cellular uptake and ameliorate the cytotoxicity induced by the chemotherapeutic agents including cisplatin. Also, recent studies had revealed that the increased levels of nitric oxide in the inner ear were associated with increased expression of the antioxidant enzymes with decreased formation of the proinflammatory cytokines (Eisenhut, 2019; Fujimoto and Yamasoba, 2019). Also, the reported cross-talk between nitric oxide and TGF-β1 was suggested to play a crucial role in a wide range of human diseases including ototoxicity (Vodovotz et al., 2004). L-arginine is an amino acid that plays a key role in the synthesis of body proteins. It is found in protein-rich foods such as red meat, fish, soya beans, whole grains and dairy products (Dashtabi et al., 2015). Being the main source of nitric oxide in the body, studying the effect of L-arginine on cisplatin-induced ototoxicity may be of particular interest (Mahran et al., 2011). The aim of this study was to explore the effect of L-arginine on cisplatin-induced ototoxicity in rats.

## Materials and Methods

*Drugs and reagents*

Cisplatin was obtained as solution vials from Mylan, Greece (1 mg/ml). L-arginine was obtained from Sigma-Aldrich Co., St. Louis, Missouri, USA (CAS Number 74-79-3) and dissolved in normal saline. All other chemicals and reagents were purchased from Sigma-Aldrich Co. (St. Louis, Missouri, USA).

*Experimental animals*

In this study, we used thirty adult male Wistar rats weighing about 140–180 g obtained from the animal house of Tanta University, Egypt. They were allowed to acclimatize for two weeks before starting the experiment. The animals were kept in a special room at a constant temperature of 25 ± 3% with relative humidity of 56 ± 3% and exposed to 12 h light/dark cycle. They were fed with standard diet and water provided ad libitum. All animal experiments were complied with the ARRIVE guidelines and were carried out in accordance with the U.K. Animals (Scientific Procedures) Act, 1986 and associated guidelines, EU Directive 2010/63/EU for animal experiments. This study was approved by the Research Ethics Committee of Faculty of Medicine, Tanta University, Egypt. 

*Study design*

All rats included in this study were confirmed to have hearing ability within the normal range by measurement of the auditory brainstem response (ABR). Briefly, rats were anesthetized using a mixture of ketamine in a dose of 40 mg/kg and xylazine in a dose of 10 mg/kg and kept warm with a heating pad. ABR was measured using TDT system II (Tucker Davis Technologies, Gainesville, FL) and the standard modular system with TDT BIO SIG software. An active electrode lead was inserted at the vertex, the reference electrode was inserted at the test ear and the ground electrode was inserted below the neck muscle. Auditory stimuli were determined in response to 100-μs clicks and 10-ms tone burst with a rise/fall time of 1 ms at frequencies of 4, 8, 16, and 32 kHz. The sound intensity was progressively decreased in 5-dB steps, and the ABR threshold was defined as the lowest stimulus intensity that produced a replicable waveform response (Akil et al., 2016). After that, rats were randomly divided into 3 equal groups as follows:

a) Control group; received a single intraperitoneal injection of normal saline. 

b) Cisplatin group; received a single intraperitoneal injection of cisplatin in a dose of 14 mg/kg body weight (Astolfi et al., 2016).

c) Cisplatin + L-arginine group; received L-arginine orally in a dose of 100 mg/kg body weight daily for seven days before and continued for 3 days after cisplatin injection (Adejare et al., 2020).

Three days after cisplatin injection, rats were anaesthetized for measurement of ABR. Then, all animals were decapitated and the temporal bone was removed. The otic capsule was opened and the cochlea was isolated by microdissection in a standard extracellular solution composed of 142 mM sodium chloride, 5.37 mM potassium chloride, 1.47 mM magnesium chloride, 2 mM calcium chloride, 300 mOsm with pH 7.2. The tissues of the right cochlea were homogenized then centrifuged at 3,000 rpm for 15 min and the supernatant was used for determination of the biochemical parameters. The tissues of the left cochlea were subjected to histopathological and electron microscopic examination. 

*Determination of tissue oxidative stress parameters*

Tissue malondialdehyde (MDA) levels were determined according to Ohkawa et al. (1979). Tissue glutathione reductase (GR) was estimated using ELISA kits purchased from Creative Diagnostics Co., USA (Catalog # DEIA-BJ2143) according to the manufacturer’s protocol. Tissue glutathione-S-transferase (GST) was measured using ELISA kits supplied by MyBioSource, Inc., San Diego, CA, USA (Catalog # MBS260372) according to the manufacturer’s protocol.

*Determination of tissue total nitrate/nitrite, nuclear factor (erythroid-derived 2)-like 2 (Nrf2) and heme oxygenase-1 (HO-1) content*

Total nitrate/nitrite was measured in the cytosolic fraction of the cochlear tissues as an indicator to nitric oxide production according to Miranda et al., (2001). Tissue Nrf2 content was measured using ELISA kits purchased from Creative Diagnostics Co., USA (Catalog # DEIA-XYA1347) according to the manufacturer’s instructions. Tissue HO-1 content was measured using ELISA kits obtained from Aviva Systems Biology, San Diego, CA, USA (Catalog # OKDD00602) according to the manufacturer’s protocol. The total protein content was estimated according to Lowry et al., (1951).

*Determination of tissue tumor necrosis factor alpha (TNF-α), interleukin-15 (IL-15), and transforming growth factor beta 1 (TGF-β1)*

Tissue TNF-α was determined using rat ELISA kits purchased from RayBiotech, Inc., USA (Catalog # ELR-TNFa-1) according to the manufacturer’s instructions. Tissue TGF-β1 was assessed using rat ELISA kits obtained from Thermo Fisher Scientific, Inc., Germany (Catalog # BMS623-3) according to the manufacturer’s protocol. Tissue IL-15 was measured using rat ELISA kits supplied by MyBioSource, Inc., San Diego, CA, USA (Catalog # MBS701942) according to the protocol of the manufacturer. 

*Histopathological examination of the cochlear tissues*

Immediately after extraction, the tissues of the left cochlea were fixed in 4 % paraformaldehyde solution (pH 7.4) for 48 h at 4°C. Then, these specimens were decalcified by immersion in 0.1 mol/L ethylene diamine tetra acetic acid (EDTA) for one week. The decalcified tissues were then processed using the routine histological methods and embedded in paraffin blocks. The samples were cut on the perimodiolar plane (At a thickness of 5 μm) using a rotary microtome. Every third section was mounted on a glass slide and stained with haematoxylin and eosin and examined under light microscope (Habybabady et al., 2018). Image analysis was performed by image analysis software program (Image J. version 1.39 p, Java 1.6) which was used to measure the mean count of spiral ganglia in all groups. 

*Scanning electron microscopic examination of the cochlear tissues*

 The cochlear tissues were fixed in 3% glutaraldehyde /0.1 M phosphate buffered saline (PBS) for one hour at room temperature. Then, specimens were rinsed in 0.1 M PBS, post-fixed in 1% OsO4 for 30 min and dehydrated through a graded series of ethanol (70^100%) for 20 min. Then, the cochlear tissues were critical-point dried, mounted on brass studs using double adhesive tape and sputter coated with 20 nm layer of gold in a JFC-ion sputter. Examination of the specimens was carried out by JEOL JSM -IT 200 scanning electron microscope at Faculty of Science, Alexandria University, Egypt.

*Statistical analysis*

Results were statistically analyzed using the statistical package for the social sciences (SPSS) version 21.0 (IBM Corp., Armonk, NY, USA). Multiple comparisons were performed using two-way analysis of variance (ANOVA) followed by Tukey-Kramer multiple comparison test. Data were represented as mean ± standard error of mean (SEM). Differences between the means of the different groups were considered statistically significant when p-value was less than 0.05.

## Results

*L-arginine ameliorated cisplatin-induced hearing loss in rats*

**Figure 1:**
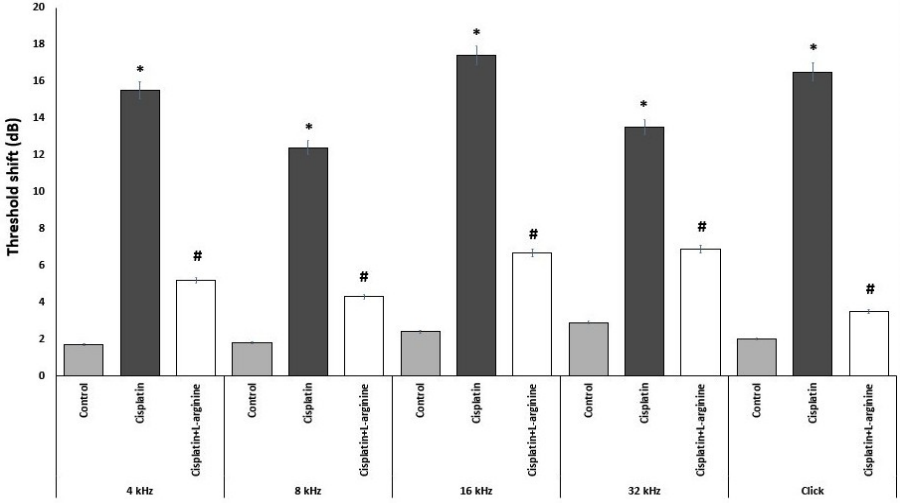
Effect of Different Treatments on the Threshold Shift in the Studied Groups. * Significant compared to the control group (p-value less than 0.05); # Significant compared to cisplatin group (p-value less than 0.05).

Cisplatin administration induced significant increase in the average ABR threshold shifts at all frequencies (4, 8, 16, and 32 kHz tone-burst and click) compared to the control group which confirms hearing loss. L-arginine given to cisplatin-treated rats induced significant decrease in the average ABR threshold shifts at all frequencies compared to cisplatin group. This indicated that pretreatment with L-arginine was able to ameliorate cisplatin-induced hearing loss in rats (Figure 1).

*Effect of different treatments on tissue antioxidant status in rats*

Cisplatin induced significant decrease in tissue GR and GST associated with significant increase in tissue MDA compared to the control group. Administration of L-arginine to cisplatin-treated rats induced significant increase in tissue GR and GST associated with significant decrease in tissue MDA compared to rats treated with cisplatin alone (Table 1).

*Effect of different treatments on tissue total nitrate/nitrite and Nrf2/HO-1 content in rats*

Cisplatin induced significant decrease in tissue total nitrate/nitrite and Nrf2/HO-1 content compared to the control group. Administration of L-arginine to cisplatin-treated rats induced significant increase in tissue total nitrate/nitrite and Nrf2/HO-1 content compared to rats treated with cisplatin alone (Table 2). 

*Effect of different treatments on tissue TGF-β1, IL-15 and TNF-α in rats*

There was significant increase in tissue TGF-β1, IL-15 and TNF-α in rats treated with cisplatin compared to the control group. Administration of L-arginine to cisplatin-treated rats induced significant decrease in tissue TGF-β1, IL-15 and TNF-α compared to rats treated with cisplatin alone (Table 3).

*Histopathological results*

**Figure 2:**
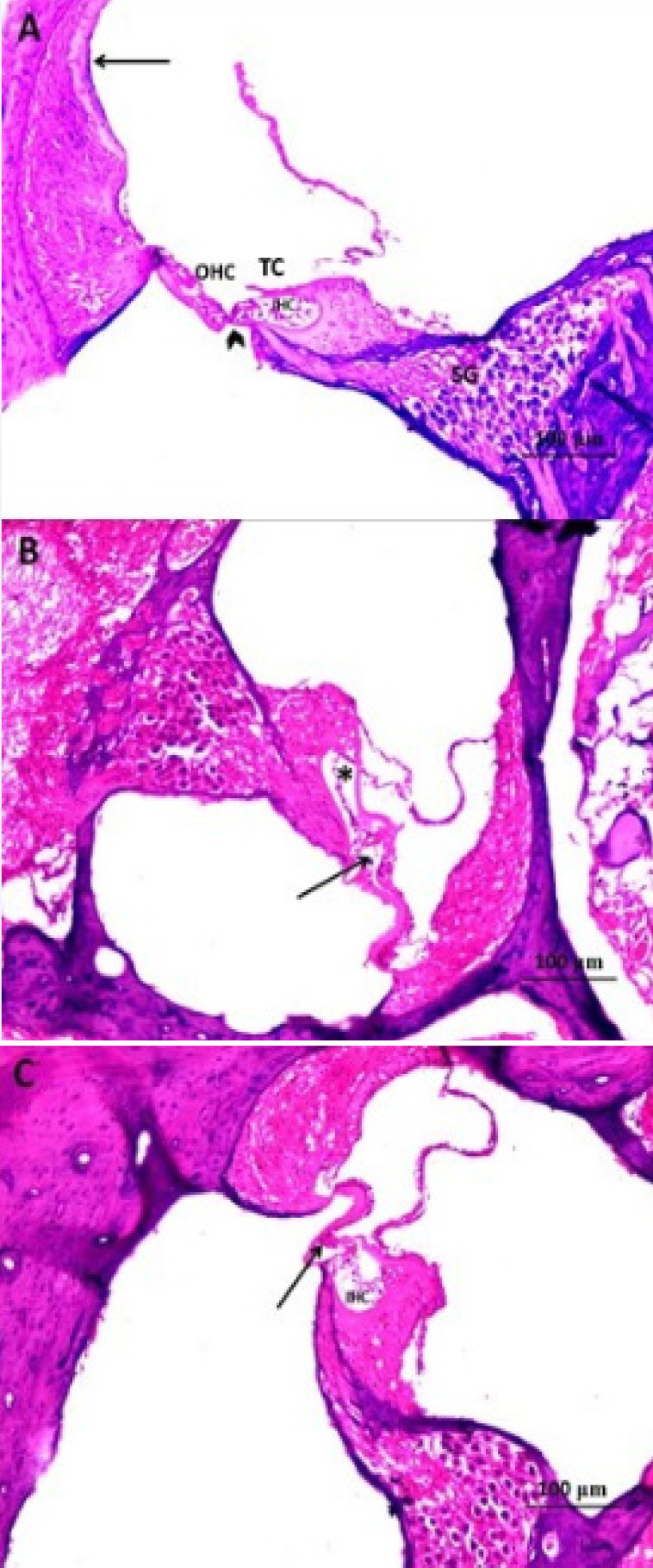
A Photomicrograph of Rat Cochlea of A) control group showing normal appearance and histology of cochlea, the organ of Corti is seen resting on the basilar membrane (arrow head). Three rows of outer hair cells (OHC) and one row of inner hair cells (IHC) are seen covered with the tectorial membrane (TC). The stria vascularis (Arrow) and spiral ganglia (SG) are clearly visible; B) cisplatin treated group showing disorganised architecture of IHCs (*) and disappearance of most OHCs (Arrow); C) cisplatin + L-arginine group showing preserved normal appearance of IHCs (ICH) and partially preserved architecture of OHCs (Arrow) (H&E x200).

**Figure 3:**
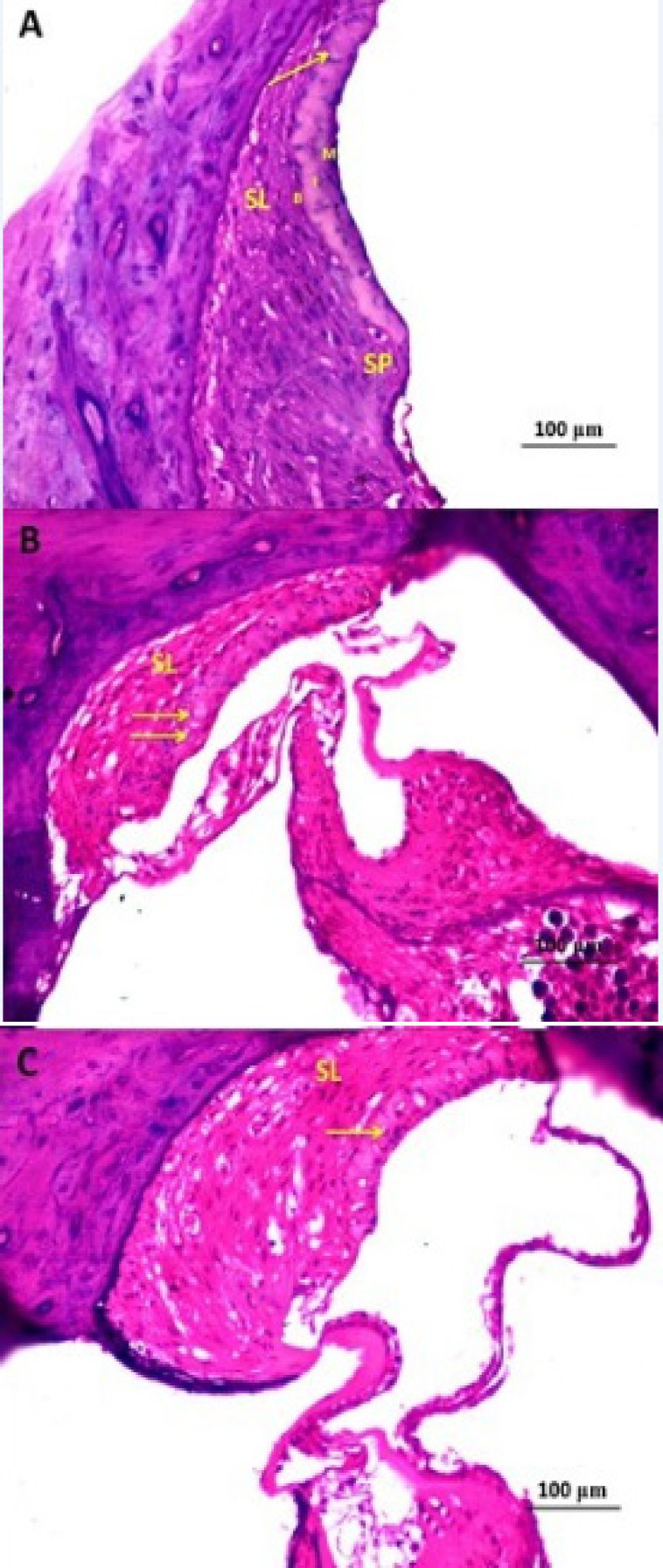
A Photomicrograph of Rat Cochlea of A) control group showing stria vascularis epithelium composed of three layers of cells; outer marginal (M), intermediate (I) and basal (B) cells. The intraepithelial blood capillaries (Arrow), the spiral ligament (SL) and spiral prominence (SP) are clearly seen; B) cisplatin-treated group showing many dilated blood capillaries (Arrows) within the stria vascularis especially near to the spiral prominence with apparent decrease in the thickness of the spiral ligament (SL) as compared to the control group; C) cisplatin + L-arginine group showing restoration of the normal structure of stria vascularis (Arrow) and spiral ligament (SL) (H&E x400).

**Figure 4:**
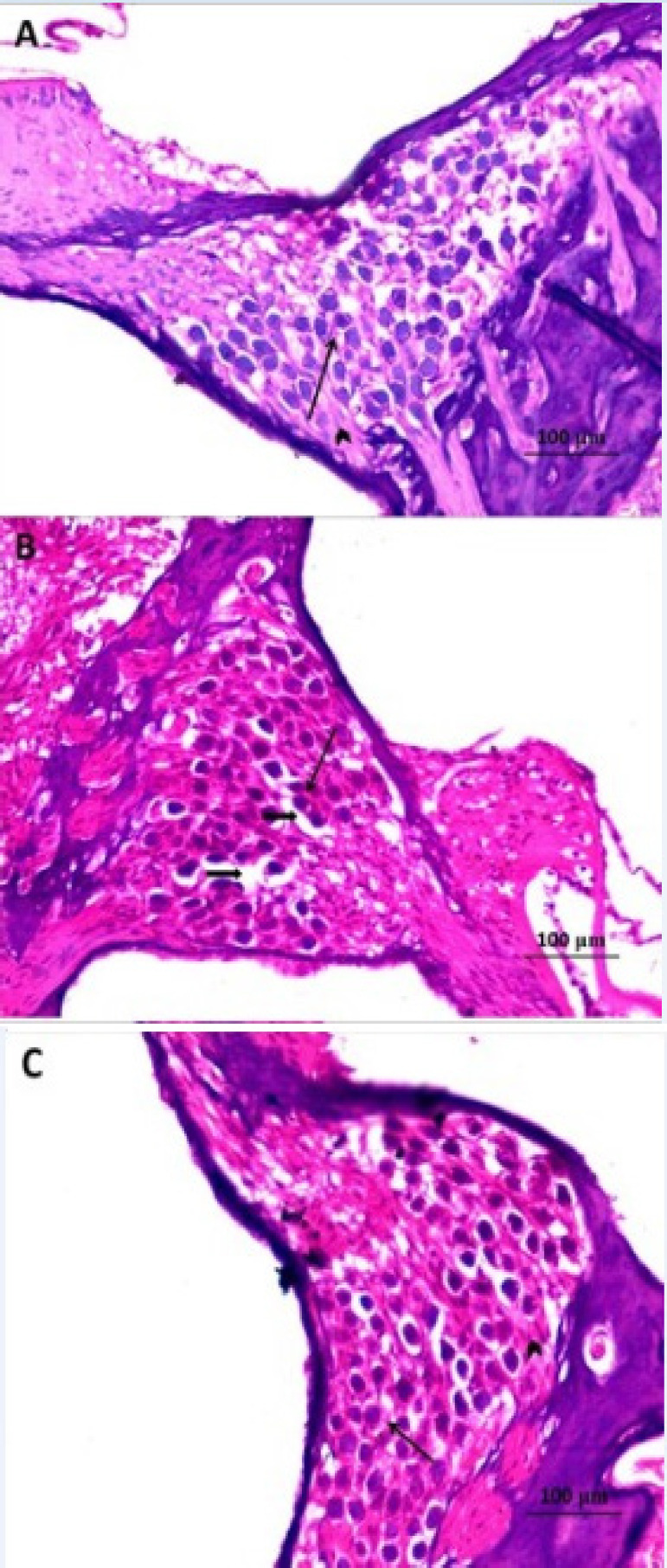
A Photomicrograph of Rat Cochlea of A) control group showing Rosenthal’s canal densely packed with spiral ganglion cells (Arrow) and fascicles of auditory nerve fibres (Arrow head ); B) cisplatin-treated group showing reduced density of spiral ganglion cells (Arrow) with some areas of focal loss (Thick arrows); C) cisplatin + L-arginine group showing apparently normal structure of the spiral ganglion cells (Arrow) and fascicles of the auditory nerve fibres (Arrow head) when compared to the control group (H&E x400)

Cisplatin administration induced significant disorganization of the architecture of the inner hair cells (IHCs) with disappearance of most outer hair cells (OHCs) (Figure 2B) associated with marked dilatation of the blood capillaries within the stria vascularis especially near to the spiral prominence with apparent decrease in the thickness of the spiral ligament compared to the control group (Figure 3B). Also, cisplatin induced significant reduction in the density of the spiral ganglion cells with focal areas of necrosis (Figure 4B). These changes were significantly ameliorated with administration of L-arginine with apparently normal architecture of IHCs and OHCs (Figure 2C) with restoration of the normal structure of the stria vascularis and spiral ligament (Figure 3C) and apparently normal spiral ganglion cells and fascicles of the auditory nerve fibres (Figure 4C). 

*Effect of different treatments on the scanning electron microscopic picture in rats*

**Figure 5:**
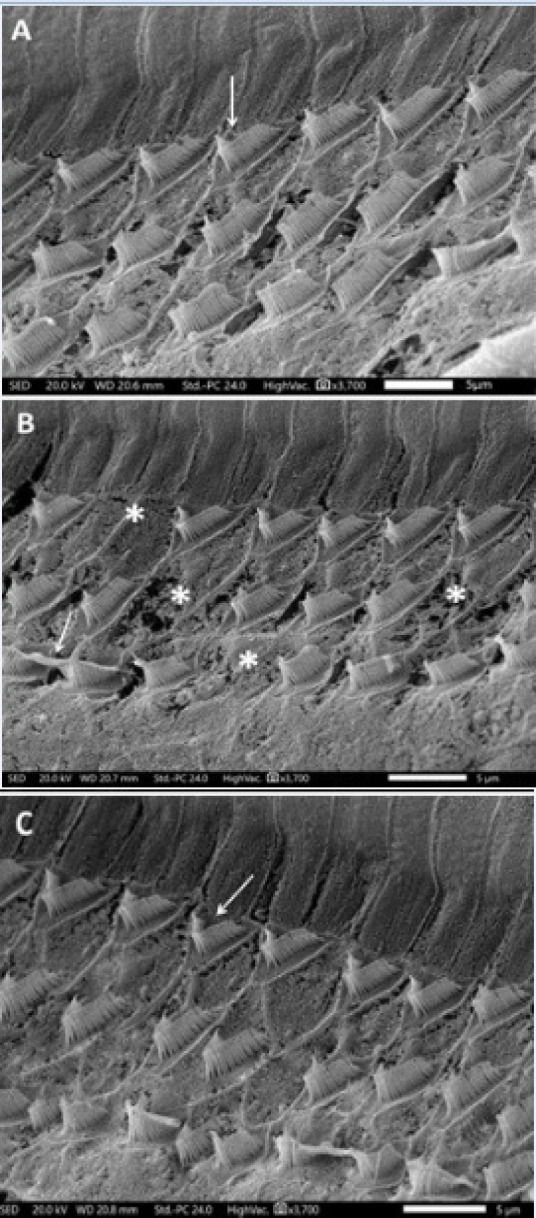
A Scanning Electron Micrograph of Rat Cochlea of A) control group showing regular arrangement (Arrow) of most of the stereocilia of the outer hair cells (OHCs); B) cisplatin treated group showing clearly visible damage in the form of areas of complete loss of stereocilia of the OHCs in the first, second and third rows (*). Also, fusion and disorganization of some stereocilia can be noticed (Arrow); C) cisplatin + L-arginine group showing regular arrangement of most of the stereocilia of the outer hair cells (OHCs) (Arrow) (SEM x 3,700 ).

**Figure 6:**
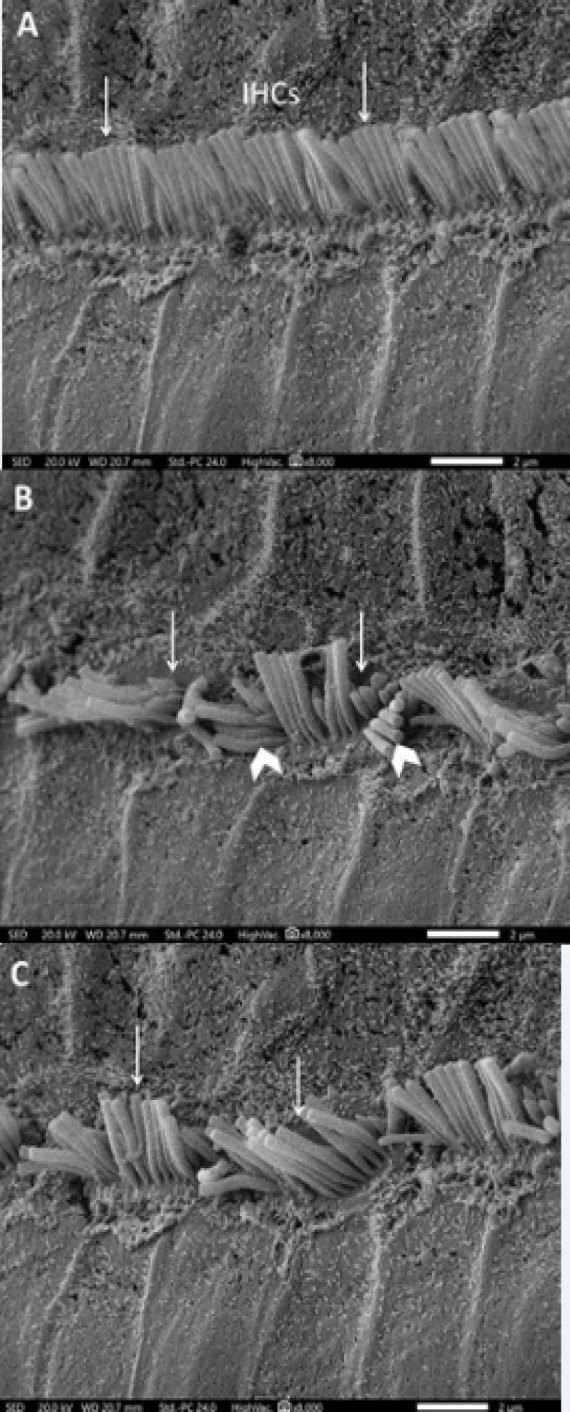
A Scanning Electron Micrograph of Rat Cochlea of A) control group showing regular arrangement (Arrows) of most of the stereocilia of the inner hair cells (IHCs); B) cisplatin treated group showing clearly visible damage in the form of shortening of some sterocillia of the inner hair cells (IHCs) (Arrows). Also, disorganization of some stereocilia can be noticed (Arrow heads); C) cisplatin + L-arginine group showing regular arrangement of most of the stereocilia of the inner (IHCs) (Arrows) (SEM x 8,000).

Administration of cisplatin induced cochlear damage in the form of areas of complete loss of stereocilia of OHCs in the first, second and third rows (Figure 5B) and fusion and disorganization of some stereocilia of IHCs (Figure 6B). Administration of L-arginine to cisplatin-treated rats resulted in restoration of the regular arrangement of most of the stereocilia of OHCs (Figure 5C) and IHCs (Figure 6C).

**Table 1:** Effect of Different Treatments on Tissue Glutathione Reductase (GR), Glutathione-S-Transferase (GST) and Malondialdehyde (MDA) in the Studied Groups (mean ± SEM)

	Control	Cisplatin	Cisplatin + L-arginine
Tissue GR (U/g/min)	212.3 ± 11.4	126.81 ± 6.8*	164.7 ± 7.93^#^
Tissue GST (U/mg protein)	33.24 ± 1.63	15.35 ± 0.72*	24.41 ± 1.29^#^
Tissue MDA (µmol/g tissue)	109.4 ± 4.8	265.7 ± 10.24 *	183.7 ± 8.65 ^#^

*, Significant compared to the control group (p-value less than 0.05); ^# ^, Significant compared to cisplatin group (p-value less than 0.05)

**Table 2:** Effect of Different Treatments on Tissue Total Nitrate/Nitrite, Nuclear Factor (erythroid-derived 2)-like 2 (Nrf2) and Heme Oxygenase-1 (HO-1) Content in the Studied Groups (mean ± SEM)

	Control	Cisplatin	Cisplatin + L-arginine
Tissue total nitrate/nitrite (% of control)	100 ± 6.26	62.1 ± 3.51*	82.3 ± 4.93^#^
Tissue HO-1 (ng/mg tissue)	0.25±0.02	0.08±0.01*	0.17±0.01^#^
Tissue Nrf2 (x10^-1^ ng/mg)	0.32±0.03	0.13±0.01*	0.24±0.02^#^

*, Significant compared to the control group (p-value less than 0.05); ^#^ , Significant compared to cisplatin group (p-value less than 0.05)

**Table 3:** Effect of Different Treatments on Tissue Tumor Necrosis Factor Alpha (TNF-α), Interleukin-15 (IL-15), and Transforming Growth Factor Beta 1 (TGF-β1) in the Studied Groups (mean ± SEM)

	Control	Cisplatin	Cisplatin + L-arginine
Tissue TNF-α (pg/ mg protein)	37.2 ± 2.1	527.3 ± 19.91*	203.5 ± 11.12 ^#^
Tissue IL-15 (pg/ mg protein)	145.2 ± 7.4	722.3 ± 25.6*	342.6 ± 15.31^ #^
Tissue TGF-β1 (pg/ mg protein)	23.42 ± 1.51	96.2 ± 5.72*	59.14 ± 3.13 ^#^

* Significant compared to the control group (p-value less than 0.05); ^#^, Significant compared to cisplatin group (p-value less than 0.05)

## Discussion

Ototoxicity is one of the drawbacks that may occur during treatment of the malignant tumors with cisplatin (Sheth et al., 2017). Up till now, the exact mechanisms that underlie cisplatin ototoxicity are still unclear. Among the proposed mechanisms, oxidative stress was incriminated to play a major role in the pathogenesis of cisplatin-induced ototoxicity (Gozeler et al., 2019). Cisplatin was reported to increase generation of reactive oxygen species (ROS) either by stimulating the enzyme systems linked to this process or by decreased expression of the antioxidant enzymes. The increased ROS often lead to the deleterious effects of oxidative stress on various body tissues, including the inner ear (Kilic et al., 2019). Also, cisplatin was proven to covalently bind to the sulfhydryl groups within the antioxidant enzymes, leading to their inactivation (Sheth et al., 2017). Moreover, cisplatin administration may lead to depletion of the metal cofactors, such as copper and selenium, which are essential for the activity of many antioxidant enzymes such as glutathione peroxidase and superoxide dismutase (Chen and Kuo, 2010). This was in agreement with the results of the present study where cisplatin induced significant decrease in the activities of GR and GST in the cochlear tissues with significant increase in tissue MDA levels compared to the control group.

In the present study, administration of L-arginine to cisplatin-treated rats leads to restoration of the antioxidant enzyme activities with significant decrease in tissue MDA levels compared to rats treated with cisplatin alone. This was in the same line with Shan et al., (2013) who attributed these effects to the antioxidant properties of L-arginine. Fazelian et al., (2014) reported that L-arginine can be considered as an effective free radical scavenger in various body tissues. Also, Abu-Serie et al., (2015) demonstrated that daily supplementation with L-arginine can increase the activities of some antioxidant enzyme in patients with chronic diseases.

Nitric oxide was reported to play a vital role in amelioration of cisplatin-induced cochlear toxicity (Jamesdaniel et al., 2016). Wong and Lerner (2015) demonstrated that nitric oxide synthase inhibitors lead to significant reduction in tissue nitric oxide levels which in turn leads to significant decrease in the activities of the antioxidant enzymes. On the other hand, nitric oxide donors such as L-arginine were proven to ameliorate nitrosative and oxidative stress generated by cisplatin in various body tissues (Saad et al., 2002). This was in accordance with the results of the present study where L-arginine was able to restore total nitrate/nitrite levels in the cochlear tissues which were correlated with amelioration of the effects of oxidative stress induced by cisplatin administration.

Recent reports revealed the important role of Nrf2/HO-1 pathway in the pathophysiology of cisplatin-induced cochleotoxicity (Kim et al., 2009). Nrf2 and its downstream enzymes such as HO-1 were proven to protect the cells from oxidative stress induced by cisplatin via regulation of the intracellular redox status (Lee et al., 2019). Nrf2 knockout mice were reported to be more sensitive to the injury induced by ROS in the pulmonary tissues (Vomund et al., 2017). In addition, Nrf2/HO-1 pathway was proven to regulate the inflammatory processes in various body tissues (Kim et al., 2009). Agents that inhibit Nrf2/HO-1 pathway were able to increase the expression of the proinflammatory cytokines and activate the inflammatory cascade (Subedi et al., 2019). Overexpression of wild-type Nrf2 was able to attenuate cisplatin-induced pro-inflammatory cytokines production, whereas dominant negative Nrf2 didn’t produce this effect (Kim et al., 2015). This was in the same line with the results of the present study where cisplatin induced significant decrease in Nrf2/HO-1 content compared to the control group which was associated with decreased activity of the antioxidant enzymes and increased expression of the proinflammatory cytokines.

In the present study, the antioxidant and the anti-inflammatory effects of L-arginine may be partially attributed to activation of Nrf2/HO-1 pathway (Liang et al., 2018). Tong and Zhou (2017) reported that L-arginine has the ability to antagonize the effects of renal ischemia/reperfusion injury in rats through regulation of nuclear factor kappa B (NF-κB) and Nrf2/HO-1 signaling cascades. Also, the increased expression of Nrf2 induced by L-arginine was reported to regulate the adaptive responses that protect various tissues against oxidative stress (Liang et al., 2018).

TGF-β1 signaling was proven to play a crucial role in the pathogenesis of cisplatin-induced ototoxicity (Feghali et al., 2001). Cisplatin was reported to increase TGF-β1 production which regulates NF-κB dependent gene expression in the cochlear tissues (So et al., 2007). These effects may lead to subsequent increase in the production of the proinflammatory cytokines with the resulting damage to the hair cells (Murillo-Cuesta et al., 2015). Also, TGF-β1 is a central mediator of fibrogenesis in the inner ear. Increased expression of TGF-β1 in the cochlear tissues induced by cisplatin was incriminated to be responsible for replacement of the hair cells with fibrous tissue (Bas et al., 2020). This was in agreement with the results of the present study where cisplatin induced significant increase in TGF-β1 production in the cochlear tissues associated with significant increase in tissue TNF-α and IL-15 with significant disorganization of the architecture of the cochlear tissues compared to the control group.

In the present study, L-arginine given to cisplatin-treated rats was able to decrease the levels of TGF-β1 in the cochlear tissues with subsequent decrease in the levels of TNF-α and IL-15 and alleviated the inflammatory changes in the histopathological and electron microscopic pictures compared to rats treated with cisplatin alone. This was in the same line with the results of Meng et al., (2017) who attributed these effects to the anti-inflammatory properties of L-arginine together with its ability to affect TGF-β1/NF-κB crosstalk which is proposed to be the main regulator of the inflammatory cascade. 

In conclusion, L-arginine may serve as a promising therapeutic modality for amelioration of cisplatin-induced ototoxicity. This may be due to its ameliorative effects on oxidative stress, inflammation and TGF-β1 signaling together with restoration of tissue total nitrate/nitrite levels and Nrf2/HO-1 content. These effects were reflected on improvement of the histopathological and electron microscopic picture of the cochlear tissues. Further studies are needed to explore the exact molecular and cellular mechanisms that may underlie these effects and the possibility of clinical application of these findings.

## References

[B1] Abu-Serie MM, El-Gamal BA, El-Kersh MA, El-Saadani MA (2015). Investigation into the antioxidant role of arginine in the treatment and the protection for intralipid-induced non-alcoholic steatohepatitis. Lipids Health Dis.

[B2] Achkar IW, Abdulrahman N, Al-Sulaiti H (2018). Cisplatin based therapy: the role of the mitogen activated protein kinase signaling pathway. J Transl Med.

[B3] Adejare A, Oloyo A, Anigbogu C, Jaja S (2020). l-arginine supplementation increased only endothelium-dependent relaxation in sprague-dawley rats fed a high-salt diet by enhancing abdominal aorta endothelial nitric oxide synthase gene expression. Clin Med Insights Cardiol.

[B4] Akil O, Oursler AE, Fan K, Lustig LR (2016). Mouse auditory brainstem response testing. Bio Protoc.

[B5] Astolfi L, Simoni E, Valente F (2016). Coenzyme Q10 plus multivitamin treatment prevents cisplatin ototoxicity in rats. PLoS One.

[B6] Bas E, Anwar MR, Van De Water TR (2020). TGF β-1 and WNT signaling pathways collaboration associated with cochlear implantation trauma-induced fibrosis. Anat Rec (Hoboken).

[B7] Chen HH, Kuo MT (2010). Role of glutathione in the regulation of Cisplatin resistance in cancer chemotherapy. Met Based Drugs.

[B8] Dasari S, Tchounwou PB (2014). Cisplatin in cancer therapy: molecular mechanisms of action. Eur J Pharmacol.

[B9] Dashtabi A, Mazloom Z, Fararouei M, Hejazi N (2015). Oral L-Arginine administration improves anthropometric and biochemical indices associated with cardiovascular diseases in obese patients: A randomized, single blind placebo controlled clinical trial. Res Cardiovasc Med.

[B10] Eisenhut M (2019). Evidence supporting the hypothesis that inflammation-induced vasospasm is involved in the pathogenesis of acquired sensorineural hearing loss. Int J Otolaryngol.

[B11] Fazelian S, Hoseini M, Namazi N (2014). Effects of L- Arginine supplementation on antioxidant status and body composition in obese patients with pre-diabetes: A randomized controlled clinical trial. Adv Pharm Bull.

[B12] Feghali JG, Liu W, Van De Water TR (2001). L-n-acetyl-cysteine protection against cisplatin-induced auditory neuronal and hair cell toxicity. Laryngoscope.

[B13] Fujimoto C, Yamasoba T (2019). Mitochondria-targeted antioxidants for treatment of hearing loss: A Systematic Review. Antioxidants (Basel).

[B14] Gonçalves MS, Silveira AF, Teixeira AR, Hyppolito MA (2013). Mechanisms of cisplatin ototoxicity: theoretical review. J Laryngol Otol.

[B15] Gozeler MS, Ekinci Akdemir FN, Yildirim S (2019). Levosimendan ameliorates cisplatin-induced ototoxicity: Rat model. Int J Pediatr Otorhinolaryngol.

[B16] Habybabady RH, Mortazavi SB, Khavanin A (2018). Protective effects of N-Acetyl-L-Cysteine on the density of spiral ganglion cells and histological changes induced by continuous noise exposure in rats. Malays J Med Sci.

[B17] Hara T, Yoshida E, Fujiwara Y, Yamamoto C, Kaji T (2017). Transforming growth factor-β1 modulates the expression of syndecan-4 in cultured vascular endothelial cells in a biphasic manner. J Cell Biochem.

[B18] Jamesdaniel S, Rathinam R, Neumann WL (2016). Targeting nitrative stress for attenuating cisplatin-induced downregulation of cochlear LIM domain only 4 and ototoxicity. Redox Biol.

[B19] Kilic K, Sakat MS, Akdemir FNE (2019). Protective effect of gallic acid against cisplatin-induced ototoxicity in rats. Braz J Otorhinolaryngol.

[B20] Kim SJ, Park C, Han AL (2009). Ebselen attenuates cisplatin-induced ROS generation through Nrf2 activation in auditory cells. Hear Res.

[B21] Kim SJ, Park C, Lee JN (2015). Erdosteine protects HEI-OC1 auditory cells from cisplatin toxicity through suppression of inflammatory cytokines and induction of Nrf2 target proteins. Toxicol Appl Pharmacol.

[B22] Lee J, Jung SY, Yang KJ (2019). α-Lipoic acid prevents against cisplatin cytotoxicity via activation of the NRF2/HO-1 antioxidant pathway. PLoS One.

[B23] Liang M, Wang Z, Li H (2018). l-Arginine induces antioxidant response to prevent oxidative stress via stimulation of glutathione synthesis and activation of Nrf2 pathway. Food Chem Toxicol.

[B24] Lowry OH, Rosenbrough NJ, Farr ALL, Randall RJ (1951). Protein measurement with the folin phenol reagent. J Biol Chem.

[B25] Mahran YF, Khalifa AE, El-Demerdash E (2011). A comparative study of protective mechanisms of glycine and L-arginine against cisplatin-induced nephrotoxicity in rat renal cortical slices. Drug Discov Ther.

[B26] Meng Q, Cooney M, Yepuri N, Cooney RN (2017). L-arginine attenuates interleukin-1β (IL-1β) induced nuclear factor Kappa-Beta (NF-κB) activation in Caco-2 cells. PLoS One.

[B27] Miranda KM, Espey MG, Wink DA (2001). A rapid, simple spectrophotometric method for simultaneous detection of nitrate and nitrite. Nitric Oxide.

[B28] Murillo-Cuesta S, Rodríguez-de la Rosa L, Contreras J (2015). Transforming growth factor β1 inhibition protects from noise-induced hearing loss. Front Aging Neurosci.

[B29] Ohkawa H, Ohishi N, Tagi K (1979). Assay for lipid peroxides in animal tissues by thiobarbituric acid reaction. Anal Biochem.

[B30] Rybak LP, Mukherjea D, Ramkumar V (2019). Mechanisms of cisplatin-induced ototoxicity and prevention. Semin Hear.

[B31] Saad SY, Najjar TA, Daba MH, Al-Rikabi AC (2002). Inhibition of nitric oxide synthase aggravates cisplatin-induced nephrotoxicity: effect of 2-amino-4-methylpyridine. Chemotherapy.

[B32] Schacht J, Talaska AE, Rybak LP (2012). Cisplatin and aminoglycoside antibiotics: hearing loss and its prevention. Anat Rec (Hoboken).

[B33] Shan L, Wang B, Gao G, Cao W, Zhang Y (2013). L-Arginine supplementation improves antioxidant defenses through L-arginine/nitric oxide pathways in exercised rats. J Appl Physiol.

[B34] Sheth S, Mukherjea D, Rybak LP, Ramkumar V (2017). Mechanisms of cisplatin-induced ototoxicity and otoprotection. Front Cell Neurosci.

[B35] So H, Kim H, Lee JH (2007). Cisplatin cytotoxicity of auditory cells requires secretions of proinflammatory cytokines via activation of ERK and NF-kappaB. J Assoc Res Otolaryngol.

[B36] Subedi L, Lee JH, Yumnam S, Ji E, Kim SY (2019). Anti-inflammatory effect of sulforaphane on LPS-activated microglia potentially through JNK/AP-1/NF-κB inhibition and Nrf2/HO-1 activation. Cells.

[B37] Tong F, Zhou X (2017). The Nrf2/HO-1 pathway mediates the antagonist effect of L-Arginine on renal ischemia/reperfusion injury in rats. Kidney Blood Press Res.

[B38] Vodovotz Y, Zamora R, Lieber MJ, Luckhart S (2004). Cross-talk between nitric oxide and transforming growth factor-beta1 in malaria. Curr Mol Med.

[B39] Vomund S, Schäfer A, Parnham MJ, Brüne B, von Knethen A (2017). Nrf2, the master regulator of anti-oxidative responses. Int J Mol Sci.

[B40] Warnecke A, Prenzler NK, Schmitt H (2019). Defining the inflammatory microenvironment in the human cochlea by perilymph analysis: Toward liquid biopsy of the cochlea. Front Neurol.

[B41] Wink DA, Cook JA, Christodoulou D (1997). Nitric oxide and some nitric oxide donor compounds enhance the cytotoxicity of cisplatin. Nitric Oxide.

[B42] Wong VW, Lerner E (2015). Nitric oxide inhibition strategies. Future Sci OA.

